# Commentary: The effect of probiotics on the diarrhea and constipation outcomes in children: an umbrella review of systematic reviews and meta-analyses

**DOI:** 10.3389/fnut.2026.1762445

**Published:** 2026-02-23

**Authors:** Yue Peng, Tiansheng Han, Shunqing Tang

**Affiliations:** 1 Department of Obstetrics and Gynecology, Shengjing Hospital of China Medical University, Shenyang, China; 2 School of Computer Science, Shenyang Aerospace University, Shenyang, China

**Keywords:** commentary, constipation, diarrhea, probiotics, umbrella review

We read with great interest the recent umbrella review by Wang et al., titled “*The effect of probiotics on the diarrhea and constipation outcomes in children: an umbrella review of systematic reviews and meta-analyses*” (1). The authors did a good job of summarizing a large body of evidence, and their conclusion that probiotics help with childhood diarrhea agrees with the general scientific opinion.

However, we would like to point out an important concern about the way the quantitative analysis was done in the umbrella review. A key principle of meta-analysis is that the units of analysis must be independent. In an umbrella review, these units are the individual meta-analyses. A problem arises when these meta-analyses are not independent because they often include similar studies.

Looking at Table 1 in the article, we see that many of the 35 meta-analyses cover very similar topics, populations, and interventions, with overlapping time frames. For example, a series of meta-analyses by Szajewska et al. (2–7), focused on the use of specific probiotics for treating acute gastroenteritis in children. These reviews were updated with new trials, but they still included the same randomized controlled trials (RCTs) from earlier versions. This overlap of studies leads to duplication of data across the reviews.

The problem occurs when the effect sizes from these overlapping meta-analyses are combined into a forest plot within the umbrella review. This method goes against the principle of independence because it involves counting the same primary RCTs more than once. As a result, the total sample size is inflated, which narrows the confidence intervals for the summary effect estimate. This makes the results seem more precise than they actually are and can lead to misleadingly low *p*-values, increasing the risk of a Type I error (false positive). In such cases, a single, large, high-quality RCT included in multiple meta-analyses may have too much influence on the overall outcome of the umbrella review. Guidelines for conducting umbrella reviews suggest ways to manage this overlap (8, 9). One recommendation is to use the corrected covered area (CCA) to measure how much the primary studies overlap across different meta-analyses. Additionally, when there is a lot of overlap for a particular outcome or population, it is better to present the results from just one comprehensive, methodologically sound, and up-to-date meta-analysis, rather than combining all related meta-analyses.

While the authors mentioned the general limitations of the included reviews, their analysis did not address the specific issue of overlapping primary studies. As a result, the quantitative findings should be interpreted with great caution regarding their precision and reliability. This point is important for a correct understanding of the study's results.

Beyond the issue of overlapping literature, a further concern is the clinical heterogeneity arising from the conflation of distinct disease entities. This umbrella review synthesizes evidence on the effect of probiotics on “diarrhea/constipation” without distinguishing between acute vs. chronic courses or infectious vs. non-infectious etiologies. Significant differences exist in the pathophysiology, therapeutic goals, and evidence for probiotic efficacy across conditions such as acute viral gastroenteritis, antibiotic-associated diarrhea, and chronic functional constipation. A probiotic strain proven effective for one condition may not be for another. To enhance the pertinence and clinical utility of conclusions, future evidence syntheses should prioritize subgroup analyses or separate syntheses based on these key clinical dimensions.

It is worth noting that these concerns do not undermine the overall conclusion that probiotics are generally effective, as this is supported by the consistent direction of effect across many studies. However, the specific quantitative estimates presented in this umbrella review are likely biased and their clinical application ambiguous. Addressing both overlapping studies and clinical heterogeneity is crucial for the robustness of future umbrella reviews. We hope this commentary will encourage more methodologically sound and clinically nuanced evidence synthesis.
